# Etoposide-Induced Apoptosis in Cancer Cells Can Be Reinforced by an Uncoupled Link between Hsp70 and Caspase-3

**DOI:** 10.3390/ijms19092519

**Published:** 2018-08-25

**Authors:** Dmitry V. Sverchinsky, Alina D. Nikotina, Elena Y. Komarova, Elena R. Mikhaylova, Nikolay D. Aksenov, Vladimir F. Lazarev, Vladimir A. Mitkevich, Roman Suezov, Dmitry S. Druzhilovskiy, Vladimir V. Poroikov, Boris A. Margulis, Irina V. Guzhova

**Affiliations:** 1 Laboratory of Cell Protection Mechanisms, Institute of Cytology of Russian Academy of Sciences, Tikhoretsky Ave. 4, St., Petersburg 194064, Russia; dsverchinsky@gmail.com (D.V.S.); nikotina.ad@gmail.com (A.D.N.); elpouta@yahoo.com (E.Y.K.); mikhailovaer@yandex.ru (E.R.M.); aksenovn@gmail.com (N.D.A.); vl.lazarev@gmail.com (V.F.L.); roman.suezov@gmail.com (R.S.); margulis@incras.ru (B.A.M.); 2 Engelhardt Institute of Molecular Biology, Russian Academy of Sciences, 32 Vavilova, Moscow 119991, Russia; mitkevich@gmail.com; 3 Institute of Biomedical Chemistry, Pogodinskaya str., 10, bldg. 8, Moscow 119121, Russia; dmitry.druzhilovsky@ibmc.msk.ru (D.S.D.); vvp1951@yandex.ru (V.V.P.)

**Keywords:** Hsp70, Caspase-3, apoptosis, anti-cancer drugs, biological activity prediction, PASS

## Abstract

The Hsp70 chaperone binds and inhibits proteins implicated in apoptotic signaling including Caspase-3. Induction of apoptosis is an important mechanism of anti-cancer drugs, therefore Hsp70 can act as a protective system in tumor cells against therapeutic agents. In this study we present an assessment of candidate compounds that are able to dissociate the complex of Hsp70 with Caspase-3, and thus sensitize cells to drug-induced apoptosis. Using the PASS program for prediction of biological activity we selected a derivative of benzodioxol (BT44) that is known to affect molecular chaperones and caspases. Drug affinity responsive target stability and microscale thermophoresis assays indicated that BT44 bound to Hsp70 and reduced the chaperone activity. When etoposide was administered, heat shock accompanied with an accumulation of Hsp70 led to an inhibition of etoposide-induced apoptosis. The number of apoptotic cells increased following BT44 administration, and forced Caspase-3 processing. Competitive protein–protein interaction and immunoprecipitation assays showed that BT44 caused dissociation of the Hsp70–Caspase-3 complex, thus augmenting the anti-tumor activity of etoposide and highlighting the potential role of molecular separators in cancer therapy.

## 1. Introduction

The most common anti-tumor drugs target a great variety of proteins implicated in programmed cell death. Initially they cause inhibition of the cell cycle, followed by the activation of apoptotic pathways [1]. A number of cellular systems exist to suppress the progression of apoptosis [2]. One of the most effective inhibitors of apoptosis is the 70 kDa heat shock protein Hsp70 which is known for its chaperone activity. The activity of Hsp70 arises from its ability to recognize damaged protein molecules and repair their structure or mark them for proteasomal degradation [3].

However, there are two properties of Hsp70 that can have negative effects in the environment of cancerous tumors. The first is its high abundance in many cancerous cells, which quite often means a bad prognosis for a patient [4]. Secondly, the high expression of Hsp70 in cancerous cells often correlates with reduced sensitivity to anti-cancer drugs [5,6]. While this protective effect is desirable for normal cells in response to physiological stress, clinically it impedes the development of antitumor therapeutic strategies.

It has been established in recent decades that the suppression of apoptosis by Hsp70 is carried out at a number of different stages of signaling, both before and after mitochondrial depolarization. For example, Hsp70 interferes with the stress-activated protein kinase SAPK/JNK (c-Jun N-terminal kinase) [7,8] and prevents Bax translocation [9]. The presence of Hsp70 can regulate Apoptotic Protease Activating Factor 1 (APAF-1) by preventing its oligomerization [10], prevent the recruitment of Caspase-9 to the APAF-1 apoptosome [11], and neutralize the apoptosis-inducing factor (AIF) [12]. Previously, we found that Hsp70 inhibits the activation of effector Caspase-3 and Caspase-7 in myeloid leukemia U-937 cells through the formation of complexes with the enzymes [5].

We hypothesized that weakening these complexes either by binding the complexing proteins or directly preventing complex formation could accelerate caspase cleavage. Cleavage is necessary for effective antitumor activity of chemotherapy drugs, and so the discovery of substances which can weaken the Hsp70-caspase complexes is essential in order to progress anti-cancer therapies. We used bioinformatics searches to identify such compounds and one candidate was found to specifically suppress both the substrate-binding and refolding activity of Hsp70. The aim of this study was to examine whether the inhibition of complex formation between Hsp70 and effector Caspase-3 could overcome the protective power of the chaperone and therefore sensitize tumor cells to apoptosis induced by traditional anticancer drugs.

## 2. Results

### 2.1. BT44 Inhibits Chaperone Activity of Hsp70 through Direct Binding to the Chaperone Molecule

#### 2.1.1. Identification of Candidate Compounds

**Figure 1:**
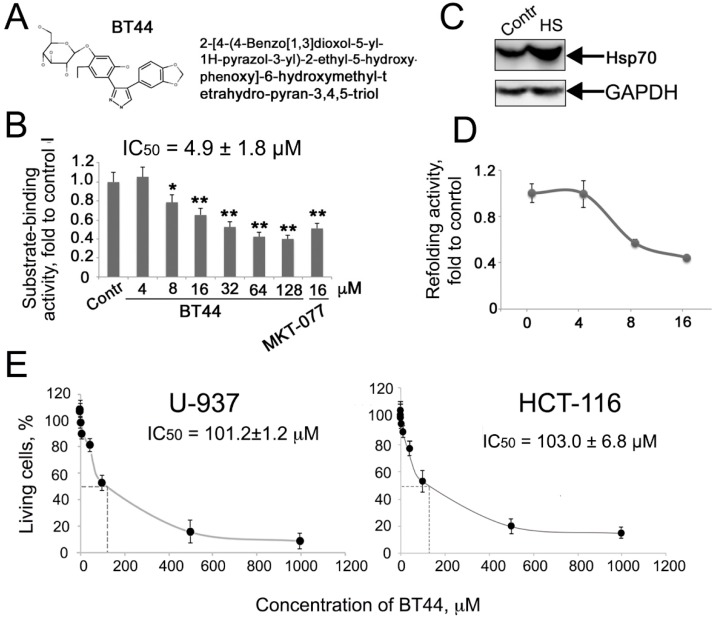
BT44 reduces chaperonic power of Hsp70 and penetrates tumor cells. (**A**) Chemical structure of [4-(4-Benzo[1,3]dioxol-5-yl-1H-pyrazol-3-yl)-2-ethyl-5-hydroxy-phenoxy]-6-hydroxymethyl-tetrahydro-pyran-3,4,5-triol, BT44; (**B**) BT44 inhibits substrate-binding activity of Hsp70. Carboxymethylated lactalbumin immobilized on wells of EIA plate was allowed to interact with pure recombinant Hsp70 in a presence of BT44 or MKT-077 and the amount of Hsp70 bound was measured with the use of a specific antibody. The representative data of four independent experiments is presented; (**C**) Western blot of HeLa cells, control and after heat shock (43 °C, 30 min) and recovered for 6 h. The lysates of HeLa cells, control or heated at 43 °C, 30 min, were subjected to gel electrophoresis and immunoblotting and the membrane was stained with anti-Hsp70 antibody. Data of three experiments is shown; (**D**) BT44 inhibits the refolding activity of Hsp70 chaperone and penetrates HeLa cells. HeLa cells were transiently transfected with a pcDNA3 plasmid-contained luciferase gene and were then heat shocked (43 °C, 30 min) to denature the enzyme in the presence of BT44 in the concentrations indicated; luciferase activity was measured in cell lysates. The representative data of three independent experiments is presented; (**E**) Effect of compound BT44 on viability of human cancer cell. U-937 and HCT-116 cells were incubated with BT44 in concentrations of 1, 5, 10, 50, 100, 500, and 1000 µM for 24 h; cell death was measured using trypan blue staining for U-937 and MTT assay for HCT-116 cells. The representative data of three independent experiments is shown.

Biological activity spectra were predicted using the PASS 2017 program for over 65,000 structural formulae from the InterBioScreen library of natural compounds, their analogs, and their derivatives. This library was selected for virtual screening of compounds compared to drug-like substances of synthetic origin [13]. The benzodioxol derivative 2-[4-(4-Benzo[1,3]dioxol-5-yl-1H-pyrazol-3-yl)-2-ethyl-5-hydroxy-phenoxy]-6-hydroxymethyl-tetrahydro-pyran-3,4,5-triol (BT44) was chosen because it was predicted to be a heat shock protein antagonist and stimulant of caspases (Table 1 and Figure 1A).

#### 2.1.2. BT44 Inhibits the Chaperone Activity of Hsp70

We first determined whether the compound was able to affect the chaperone activity of Hsp70. We estimated the concentration-dependent inhibitory capacity of BT44 in a substrate-binding assay using the well-known Hsp70 modulator MKT-077 as a reference compound [14]. In these and further experiments MKT-077 was used as an etalon compound. The binding of purified Hsp70 to a denatured protein substrate (carboxy-methylated α-lactalbumin, CMLA) was suppressed by BT44 at a minimum concentration of 8 μM, and then decreased in a dose-dependent manner (Figure 1B).

#### 2.1.3. BT44 Inhibits Hsp70 Activity in Cells

To influence the chaperone activity of Hsp70 within cancer cells, a compound must be able to enter the cell. To determine whether BT44 is able to cross cell membranes, we transiently transfected HeLa cells with a plasmid bearing the luciferase gene under a cytomegalovirus promoter. Treatment with heat shock of 43 °C for 30 min resulted in a reduction of luciferase activity by almost 100-fold in the HeLa cells. Once the temperature was reduced to 37 °C, the enzyme activity recovered spontaneously within 6 h, probably due to an accumulation of intracellular Hsp70 following heat shock (Figure 1C). The administration of BT44 prior to heat shock led to a dose-dependent decrease of re-activation, confirming that the compound penetrates to the tumor cell and inhibits the chaperone activity of Hsp70 (Figure 1D). In the control experiment, BT44 did not influence luciferase activity in non-heat shocked HeLa-luc cells, the value was equal of the control one. Importantly, BT44 did not demonstrate self-toxicity, proved by the IC_50_ of 103.0 ± 6.8 μM for HCT-116 and 101.2 ± 1.2 μM for U-937 cells (Figure 1E), as estimated by MTT assay and trypan blue staining, respectively. 

#### 2.1.4. Inhibitory Actions of BT44 Occur throuh Direct Hsp70 Binding

**Figure 2:**
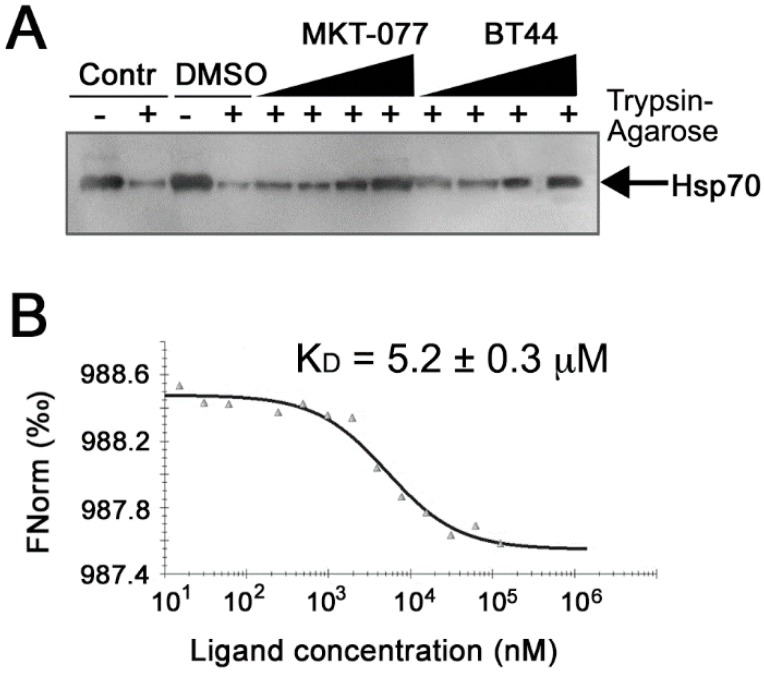
BT44 is able to bind Hsp70 molecule. (**A**) Data from DARTS assay. The products of Hsp70 trypsinolysis in the presence of BT44 and the etalon compound MKT-077, in concentrations 1, 4, 16, and 64 µM, were subjected to electrophoresis and immunoblotting using an anti-Hsp70 antibody. The data of four independent experiments is presented; (**B**) Hsp70–BT44 binding measured with the aid of microscale thermophoresis. Unlabeled BT44 (20 μM–0.61 nM) was titrated in relation to a fixed concentration of labeled Hsp70 (16 µM).

Based on this data, we hypothesized that the inhibitory actions of BT44 could result from direct binding of the compound to Hsp70. To confirm this we carried out a drug affinity responsive target stability (DARTS) assay. Western blotting confirmed that Hsp70 was digested upon incubation with trypsin-agarose beads. Both MKT-077 and BT44 prevented trypsin proteolysis in a dose-dependent manner, suggesting that both ligands are able to bind to Hsp70 in micromolar concentrations (Figure 2A).

To confirm the DARTS data we measured the affinity of BT44 to Hsp70 with the aid of microscale thermophoresis. Estimation of the affinity of BT44 for Hsp70 revealed that the *K*_D_ was 5.2 ± 0.3 µM (Figure 2B). This value indicates that the compound directly binds to Hsp70 in the micromolar range and thus reduces its substrate-binding activity.

### 2.2. BT44 Increases the Sensitivity of Cancer Cells with Over-Expressed Hsp70 to Etoposide-Induced Apoptosis

The value of Hsp70 inhibitors in cancer treatment relies on their capacity to reduce one of the main protective pathways of tumor cells and therefore increase their sensitivity to anticancer drugs.

**Figure 3:**
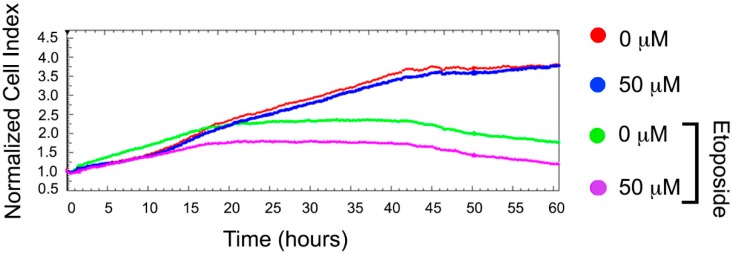
BT44 sensitizes colon HCT-116 cancer cells to etoposide. HCT-116 cells were seeded to wells of E-plates and after being attached to the bottom were incubated with BT44 in concentrations 50 μM; 2 h later 100 μM of etoposide was added. Recording with the aid of xCELLigence equipment was started immediately after drugs administration and lasted 60 h. Data from one of three independent experiments is presented.

To test this action, we used human colon carcinoma HCT-116 cells that are known to be resistant to etoposide [15]. Etoposide alone caused a decreased cell growth rate and tumor cell population after 40 h. However, the total number of tumor cells remained slightly higher than at the beginning of the recording. Pretreatment of HCT-116 cells with BT44 led to an inhibition of tumor cell growth by approximately 50% (Figure 3).

**Figure 4:**
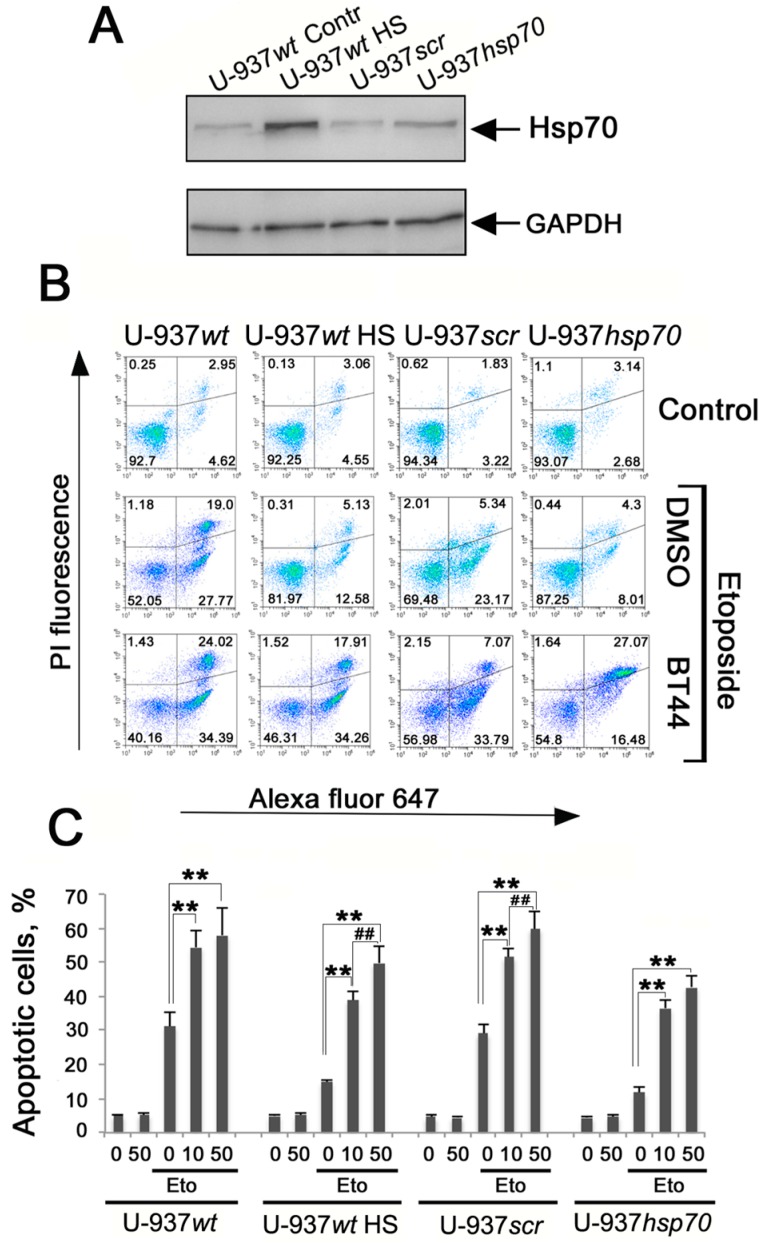
BT44 enhances the effect of etoposide in the induction of apoptosis in cancer cells. (**A**) Western blot of U-937*wt*, U-937*scr*, and U-937*hsp70* cells used for analysis. U937*wt* cells were heat shocked (43 °C, 30 min) and allowed to recover for 6 h (HS). The membrane was stained with the antibody against Hsp70. The representative data of two independent experiments is presented; (**B**,**C**) U-937*wt*, control and after heat shock (U-937*wt* HS), U-937*scr* and U-937*hsp70* were incubated with BT44 in concentrations 10 and 50 μM, and 2 h later 2 μM of etoposide was added to cell culture for 18 h. Cells were stained with Annexin-V and propidium iodide (PI) and subjected to flow cytometry analysis. (**B**) Density plots of one representative experiment; (**C**) Data is presented as the means ± standard error of the mean (SEM). A statistical difference was determined by a value of ** *p* < 0.01; ^##^ *p* < 0.01 comparing cells treated with 10 µM and 50 µM of BT44 and etoposide; the data of five independent experiments is summarized.

We predicted that BT44 would stimulate caspase activity, therefore the decline in cell growth that we observed may be due to apoptotic cell death rather than cell cycle arrest. Etoposide is known to induce apoptosis in most cancerous cells, while Hsp70 is able to arrest apoptosis at various stages of the signaling process [1]. To determine whether BT44 could stimulate apoptosis specifically in cells with high levels of Hsp70, we tested the compound on human histiocytic lymphoma U-937 cells with normal (U-937*wt* and U-937*scr*) and elevated levels of Hsp70 (U-937*wt* HS and U-937*hsp70* (Figure 4A). In line with our predictions, after etoposide administration, cells with low levels of Hsp70 (U-937*wt* and U-937*scr*) showed an almost 50% reduction in apoptotic cells compared with cells with high levels of Hsp70 (31.2 ± 4.2% vs. 15.0 ± 0.7% for U-937*wt* and U-937*wt* HS respectively, and 29.0 ± 2.7% vs. 11.7 ± 1.7% for U-937*scr* and U-937*hsp70* respectively). Pretreatment with BT44 caused a dose-dependent increase in apoptosis levels in all cell populations, with an increase of approximately 2-fold seen in cells with low levels of Hsp70 and approximately 3.5-fold seen in cells with high levels of Hsp70 (Figure 4B,C).

### 2.3. BT44 Enhances the Etoposide Sensitivity of U-937 Cells with High Hsp70 Levels

**Figure 5:**
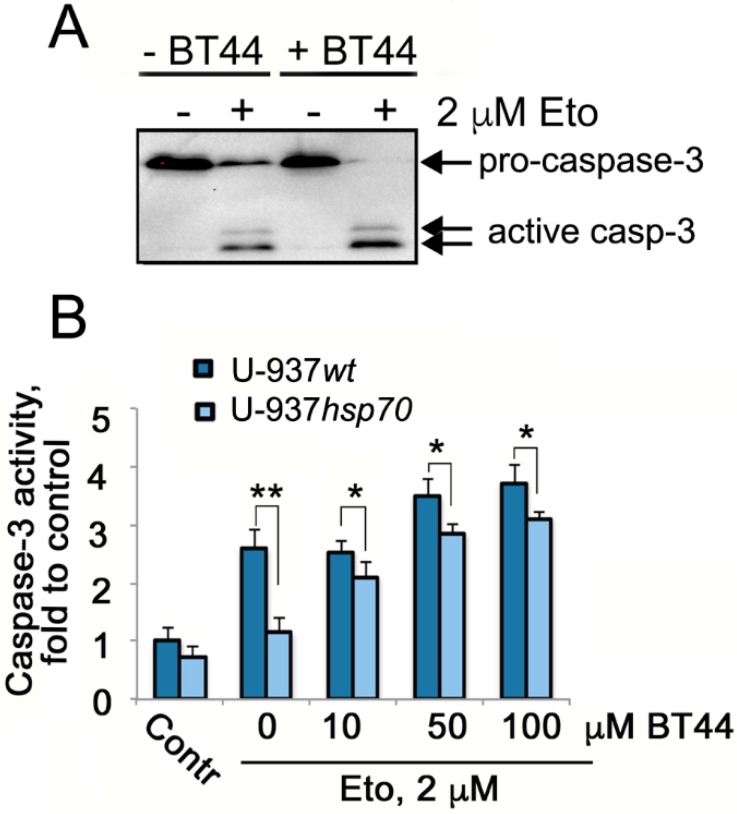
BT44 enhances Caspase-3 cleavage in U-937 cells treated with etoposide. (**A**) Western blot of U-937*wt* cells treated with BT44 and etoposide, alone or in combination. The membrane was stained with antibody against Caspase-3; (**B**) U-937*wt* and U-937*hsp70* were treated with BT44 in concentrations indicated and etoposide (2 μM), alone or in combination, and Caspase-3 cleavage was estimated with the aid of Caspase-3 enzymatic activity assay. A statistical difference was determined by a value of * *p* < 0.05, ** *p* < 0.01. The representative data of two experiments is presented.

We have previously reported that etoposide administration causes Hsp70 to bind to activated Caspase-3 in U-937 cells which over-express the chaperone [5]. Caspase-3 was more thoroughly digested when U-937*wt* cells were pretreated with BT44 (Figure 5A). Contrary to our prediction, this result indicates that BT44 does not directly stimulate Caspase-3 cleavage but enhances cleavage when it is used in combination with etoposide.

Etoposide-induced Caspase-3 cleavage in U-937*wt* and U-937*hsp70* cells treated with BT44 was further analyzed using a fluorescence-based Caspase-3 enzymatic activity assay. In lysates of cells treated with etoposide alone, the Caspase-3 cleavage was found to be 55.8% higher in U-937*wt* cells than that of U-937*hsp70* cells. Lysates of cells that had been pretreated with BT44 showed a dose-dependent increase in Caspase-3 cleavage levels. The difference between U-937*wt* and U-937*hsp70* lysates varied from 16.6% to 18.8% (Figure 5B), confirming that BT44 is able to overcome the protective action of Hsp70 in tumor cells.

### 2.4. BT44 Prevents the Binding of Hsp70 to Caspase-3

**Figure 6:**
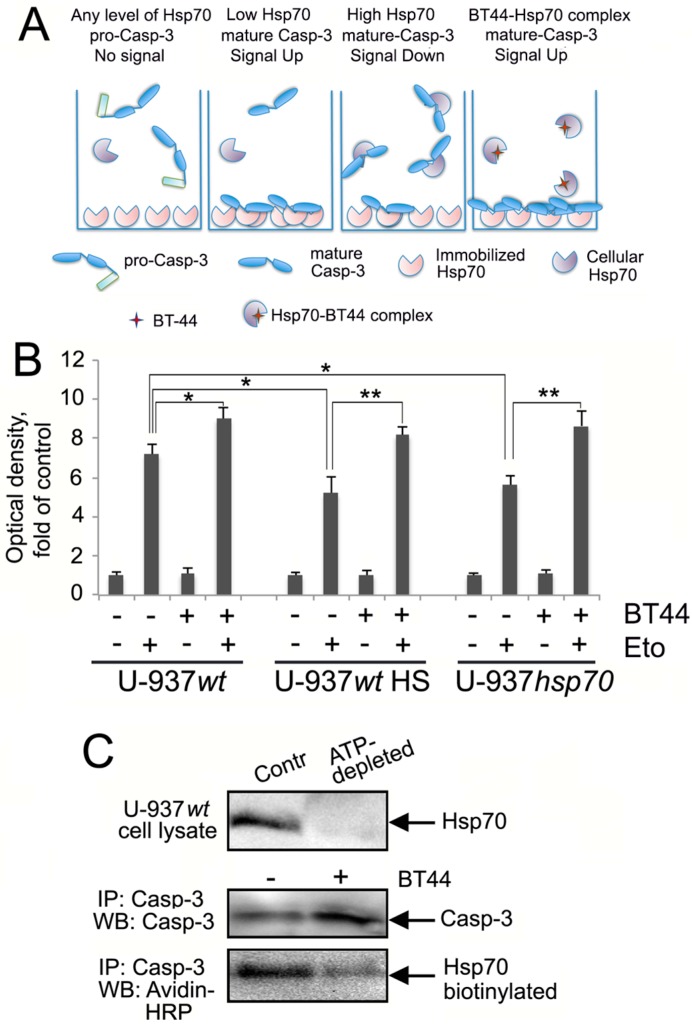
BT44 prevents binding of Hsp70 to Caspase-3 in etoposide-treated, Hsp70 over-expressing U-937 cells. (**A**) The scheme illustrating protein–protein interaction assay (Figure 6B). In cells with low levels of Hsp70 (U-937*wt* and U-937*scr*), the number of chaperone molecules was not sufficient to trap all molecules of activated Caspase-3 which bound to Hsp70 immobilized on the well surface; BT44 induced destabilization of Hsp70 and Caspase-3 complex and made the enzyme molecules to stick to Hsp70 more efficiently, and signal was elevated. When cellular Hsp70 was overexpressed (U-937*wt* after HS and U-937*hsp70*), more molecules of activated Caspase-3 were bound to cellular Hsp70, less Caspase-3 was trapped by the immobilized chaperone, and signal decreased. Cellular Hsp70 was inactivated in the presence of BT44 so the number of activated Caspase-3 molecules available for trapping with the immobilized Hsp70 was elevated; (**B**) Competitive protein–protein interaction assay data. U-937*wt*, control and after heat shock (43 °C, 30 min, 6 h of recovery), and U-937*hsp70*, were treated with 50 µM BT44 and 1 µM etoposide, alone or in combination and the cell lysates were used for analysis. The plate was then incubated for 3 h. The data of four independent experiments is shown; A statistical difference was determined by a value of * *p* < 0.05, ** *p* < 0.01; (**C**) U-937*wt* cells were treated with etoposide and 4 h later Hsp70 was depleted from cell lysate with the aid of ATP-agarose. After immunoprecipitation with anti-Caspase-3 antibody, gel slurry with Protein G-anti-Caspase-3 antibody and Caspase-3 was transferred to tubes containing pure biotinylated Hsp70 pretreated or not with BT44, and the gels with the proteins attached were subjected to electrophoresis and immunoblotting. The blot was stained using antibody to Caspase-3 and Avidin-peroxidase (Avidin-HRP). The data of two independent experiments is shown.

To assess whether BT44 inhibited the binding of Hsp70 to Caspase-3 we used a competitive protein–protein interaction assay (Figure 6A). The levels of Caspase-3 in cells with low levels of Hsp70 (U-937*wt*) increased by up to 125% following treatment with BT44 and etoposide (Figure 6B). The number of Caspase-3 molecules that bound to immobilized Hsp70 decreased by 35–50% in cells which had been subjected to heat shock or transfected with *hsp70* gene, compared to U-937*wt* cells treated with etoposide alone. Treatment of U-937*wt* or U-937*hsp70* cells with BT44 increased Caspase-3 binding by 42.5% compared with the lysate of heat shocked U-937*wt* cells or etoposide-treated U-937*hsp*70 cells (with no BT44 pretreatment). This confirms that BT44 had inactivated the majority of cellular Hsp70 molecules (see Figure 6A).

The next experiment was carried out to confirm the data of protein–protein interaction assay and to check the inhibitory effect of BT44 on the direct Caspase-3-Hsp70 complex formation in U-937 cells. In order to analyze the interaction exclusively between the two protein partners we first removed the whole pool of Hsp70 molecules with the aid of chromatography on ATP-Agarose (Figure 6C, upper panel) method employed to purify the chaperone from all known sources. To isolate Caspase-3 from the Hsp70-depleted cell lysate we used immunoprecipitation with the specific antibody attached to gel beads with Protein G. After careful washing, the beads carrying Caspase-3—as confirmed with the aid of western blotting (Figure 6C, middle panel)—were incubated with pure biotinylated Hsp70 with or without BT44; so in a tube there was everything needed for the reaction between two pure proteins and the potential inhibitor, BT44. The beads were analyzed with the aid of electrophoresis and western blotting using avidin-peroxidase conjugate for staining of Hsp70 bands (Figure 6C, lower band). The amount of biotinylated Hsp70 trapped by Caspase-3 in the presence of BT44 was found to be approximately 10-fold lower than when the chaperone was intact (that is, when BT44 was not used, Figure 6C). This confirms that the direct binding of Hsp70 to Caspase-3 can be prevented with BT44.

## 3. Discussion

The resistance of cancer cells to chemotherapy is often a result of the high expression of molecular chaperones which protects tumor cells from harmful exogenous factors including anticancer drugs [16]. The chaperone protein Hsp70 can interfere with many steps of the apoptotic pathway, inactivating signal messenger molecules and impeding death for hours or days, thus giving tumor cells a chance to survive and divide [1]. Preventing the binding of Hsp70 to apoptotic enzymes may be an approach to inhibit the protective mechanisms of cancer cells and so increase their sensitivity to anti-cancer drugs.

Two assays were used to confirm the direct interaction of BT44 with Hsp70, the DARTS assay and MST (Figure 2). Data from the DARTS assay showed that the proteolytic cleavage of Hsp70 was inhibited by BT44, as the chaperone exhibited increased thermodynamic stability and resistance to cleavage following treatment with Trypsin-agarose [17]. This effect was observed when the concentration of BT44 was in the micromolar range, in line with the effects of the canonical Hsp70-binder, MKT-077 (Figure 2A) [18]. This data was confirmed with the aid of MST that allowed us to estimate the *K*_D_ to be 5.2 ± 0.3 μM (Figure 2B).

To date, over 20 small-molecule inhibitors of Hsp70 have been identified and most are reported to exhibit anti-cancer activity in a variety of cell and animal models. Of these inhibitors, MKT-077 has been shown to deactivate the chaperone function of Hsp70 and inhibit the growth of human tumor cell lines [19,20]. Another Hsp70 binder, 2-phenylethynesulfonamide (PES), inhibits the chaperone by dissociating the Hsp70-substrate complexes. Treatment of tumor cells with PES leads to protein aggregation and impaired autophagy, with the result of suppression of Myc-induced lymphomagenesis [21]. Similar anti-cancer effects have been observed following administration of VER-155008, which led to reduced growth rate and apoptosis in several tumor cell lines [22]. Recently we demonstrated that the colchicine derivative AEAC binds to Hsp70, which in turn causes rat glioma C6 and mouse melanoma B16 cells to be more sensitive to doxorubicin both in vitro and in vivo [23].

We examined whether BT44 impeded the growth of colon carcinoma HCT-116 cell growth treated with etoposide (Figure 3) and promoted etoposide-induced apoptosis in human histiocytic lymphoma U-937 cells with over-expressed Hsp70 (Figure 4). Our results revealed that pretreatment with BT44 reduced the resistance of colon carcinoma cells to etoposide (Figure 3) and increased etoposide-induced apoptosis in U-937 cells with high levels of Hsp70 (U-937*wt* HS and U-937*hsp70*; Figure 4B,C). Similar growth inhibition and pro-apoptotic effects have been demonstrated in HCT-116 cell lines following modulation of the activity of the HSF1 molecular chaperone [24].

Since apoptosis is defined as caspase-dependent programmed cell death [25], we investigated whether BT44 affected Caspase-3 cleavage in U-937 cells with high and regular levels of Hsp70. Enzyme cleavage was significantly increased in U-937*wt* cells pretreated with BT44 (Figure 5A). Administered alone, BT44 did not promote the activation of Caspase-3, however it appeared to enhance the action of etoposide (Figure 5A). The discovery that cleavage of Caspase-3 was increased in cells with high Hsp70 level following BT44 treatment (Figure 5B) was particularly important, as cancer cells are often characterized by high levels of the chaperone [26].

Data from competitive protein–protein interaction and immunoprecipitation assays showed that BT44 augmented the anti-tumor activity of etoposide by dissociating the Hsp70–Caspase-3 complex (Figure 6A,B). There is little literature available relating to the application of specific disrupters of protein–protein interactions as this is a relatively new area of research. The actions of Hsp70 are facilitated by co-chaperones of the J-domain class (DNAJ) and nucleotide-exchange factors such as the Bag family proteins. The Gestwicki laboratory carried out analyses of the allosteric interaction of Bag family proteins with Hsp70 and discovered that one compound, JG-98, can inhibit the formation of Bag-3–Hsp70 complexes. This reduces the proliferative activity of tumor cells, thus suggesting that JG-98 could act as an anti-cancer drug [27].

Hsp70 can interact with several types of polypeptides, those damaged by stress, specifically binding to the chaperone-like Bag-3 and proteins, from time to time exposing their hydrophobic lacunas to Hsp70 in a manner of Caspase-3. By destroying these transient complexes with pharmacological agents we may be able to promote the release of polypeptides and therefore reduce the protective power of Hsp70 in cancer cells. These separators may therefore serve as effective assistants to classic drugs, as demonstrated by the pro-apoptotic action of etoposide in this study.

## 4. Materials and Methods

### 4.1. Virtual Screening of Compounds with Desirable Properties

We used the PASS (Prediction of Biological Activity Spectra for Substances) computer program [28] to identify compounds that could modulate the interaction of Hsp70 with its macromolecular partners. The PASS 2017 predicts several thousand kinds of biological activity with an average accuracy of about 95%. Prediction is based on the analysis of structure–activity relationships for the training set, including over one million biologically active compounds. Of the 5050 biological activities predicted by PASS 2017, the mechanisms that were considered relevant to this study are summarized in Table 2.

### 4.2. Purification of Hsp70 and Measurement of Chaperone Activity

Recombinant human Hsp70 was purified from bacteria that had been transformed with a pMSHsp70 plasmid, using a published two-step chromatography procedure as described elsewhere [29]. The substrate-binding activity of Hsp70 was measured using a modified enzyme immunoassay [23,30]. Briefly human recombinant Hsp70 (100 ng/mL) was mixed with either BT44 or MKT-077 inhibitors in a buffer that contained 20 mM Tris HCl, 20 mM NaCl, and 10 mM MgCl_2_ (pH 7.5), and then transferred to the wells. Anti-Hsp70 polyclonal antibody (RS) was added [31], followed by incubation with anti-rabbit IgG HRP-conjugated antibody (Jackson Immunochemicals, West Grove, PA, USA). After the addition of tetramethylbenzene in a citrate buffer (pH 4.5) containing hydrogen peroxide, the intensity of staining was measured using a Fluorofot “Charity” microplate reader (Probanauchpribor, St.Petersburg, Russia).

### 4.3. Cells

Human cervical carcinoma HeLa cells and human histiocytic lymphoma U-937 cells were obtained from the Russian Collection of Cell Cultures (Institute of Cytology, Russian Academy of Sciences, St. Petersburg). Cultured U-937 cells were stably transfected with pcDNA3 (U-937*scr*) or pcDNA3HSP70 (U-937*hsp70*) plasmids [5]. Human colon carcinoma HCT-116 cells were kindly provided by Dr. N. Barlev (Institute of Cytology, Russian Academy of Sciences, St. Petersburg). Both HeLa and HCT-116 cells were grown in Dulbecco’s Modified Eagle’s Medium (DMEM, Lonza, MD, USA); U-937 cells were grown in Roswell Park Memorial Institute (RPMI) medium (Lonza), with the addition of 10% fetal calf serum (Paneco, Moscow, Russia), penicillin G (100 ME/mL), and streptomycin (100 μg/mL) (Biolot, St. Peterburg, Russia). Cells were incubated in a humidified incubator at 37 °C, 6% CO_2_.

### 4.4. Refolding Assay

Cultured HeLa cells were transfected with a pcDNA3 plasmid containing the luciferase gene and analysis of refolding activity was performed following a published protocol [23].

### 4.5. Drug Affinity Responsive Target Stability

We used a published protocol [32] for the drug affinity responsive target stability (DARTS) assay, with some previously published modifications [23].

Purified Hsp70 (5 μg in 20 μL DARTS buffer, containing 50 mM Tris HCl pH 8.0, 50 mM NaCl, 10 mM CaCl_2_) was incubated with BT44 or MKT-077 (the reference substance) at a concentration of 1, 4, 16, or 64 μM at 4 °C for 1 h. Next, 2 μL of trypsin–agarose were added to each sample and the mixture incubated at 37 °C for 45 min. The gel was separated by centrifugation and the supernatant analyzed using polyacrylamide gel electrophoresis (PAGE) and immunoblotting with RS anti-Hsp70 antibodies [31] followed by anti-rabbit IgG HRP-conjugated antibody (Jackson Immunochemicals).

### 4.6. Microscale Thermophoresis

We used the NanoTemper system [33] to measure the binding capacity of BT44 to Hsp70. Purified Hsp70 was labeled with NT-647 dye through *N*-hydroxysuccinimide (NHS) coupling, according to the manufacturer’s protocol. In a typical MST experiment, the concentration of NT-647-labeled Hsp70 was kept constant (16 μM) while the concentration of unlabeled BT44 was varied between 0.61 nM and 20 μM in MST buffer (20 mM HEPES, pH 8.0, 200 mM NaCl, 1 mM β-Mercaptoethanol, 0.05% Tween-20). After a 1-h incubation at room temperature, samples were loaded into 115 premium glass capillaries and MST analysis was performed using the Monolith NT.115 (NanoTemper technology, München, Germany). The laser power was 20%. Data analysis was performed using NanoTemper Analysis software v.1.2.101.

### 4.7. Cytotoxicity Assay with xCELLigence System

The xCELLigence system (ACEA Biosciences, San Diego, CA, USA) provides noninvasive and label-free monitoring of cell viability and growth in real-time, based on the measurement of the electrical impedance of cells adhered to an electrode on the bottom of the well. Increased impedance indicates an increased number of cells adhered to the bottom at a particular time [34]. Cultured HCT-116 cells were placed into 16-well E-plates (ACEA Biosciences) at a concentration of 80,000 cells/mL. After 18 h, the cells were treated with BT44 at a concentration of 50 µM for 2 h, and then 100 µM etoposide was added to the cells. Cell proliferation was monitored for 60 h using the xCELLigence Real Time Cell Analysis (RTCA) System. Data analysis was performed using RTCA Analysis Software.

### 4.8. Detection of Apoptosis

Detection of apoptosis was performed using Annexin V-FluorTM 633 (Life Technologies, Carlsbad, CA, USA) combined with 100 μM propidium iodide (Life Technologies) staining. Untreated U-937*wt*, U-937*scr* and U-937*hsp70* cells as well as heat shocked U-937*wt* cells (43 °C, 30 min with 6 h recovery time), were treated with BT44 at a concentration of 10 or 50 μM for 6 h, either alone or in combination with 2 μM etoposide. Cells were collected after 18 h, washed in cold PBS, resuspended in the binding buffer provided by the manufacturer and stained with 5 μl Annexin-V Alexa FluorTM 647 and propidium iodide (Life Technologies) according to the manufacturer’s instructions. The percentage of apoptotic cells was then measured using the CytoFlex flow cytometer (Beckman Coulter, Brea, CA, USA) using lasers set to *λ* = 488 nm (propidium iodide) or *λ* = 638 nm (Alexa FluorTM 647) and analyzed with CytExpert 2.0 software (Beckman Coulter).

### 4.9. Western Blot Analysis

Cultured U-937*wt* cells were treated with BT44 at a concentration of 50 μM, then 2 μM of etoposide was administered 6 h later. After 15 h of incubation, cells were lysed in RIPA buffer, separated by PAGE on a 15% polyacrylamide gel and transferred onto a polyvinylidene fluoride (PVDF) membrane. The PVDF membrane was blocked with PBS containing 5% (*w*/*v*) skimmed milk and incubated with a primary antibody against Caspase-3 (Cell Signaling, Danvers, MA, USA) and secondary antibodies (Abcam, Cambridge, UK) at room temperature for 1 h. Band intensity was quantified using the ChemiDocTM system (Bio-Rad, Hercules, CA, USA). To assess the content of Hsp70 in treated cells, 20 μg of the same cell lysates were separated by PAGE on an 11.5% gel. The membrane was probed with an anti-Hsp70 monoclonal antibody (Clone 2H9) followed by a secondary anti-mouse antibody (Abcam).

### 4.10. Caspase-3 Enzymatic Activity Assay

Cultured U-937*wt* and U-937*hsp70* cells were seeded onto a 12-well plate at a concentration of 3 × 10^5^/mL. After incubation with 2 µM etoposide and 10, 50 or 100 µM BT44 for 12–14 h, cells were precipitated by centrifugation at 2000 rpm, washed twice in cold PBS and lysed in a buffer containing 100 mM HEPES, pH 7.2, 10% sucrose, 1 mM EDTA pH 8.0, 0.1% CHAPS and 10 mM DTT. Lysates were subject to two freeze-thaw cycles at −80 °C and centrifuged at 13,000× *g* for 10 min. Protein concentration in supernatant was measured with the Bradford assay, lysate containing a total of 200 µg of protein in 50 µL of lysis buffer was added into the wells of a black 96-well plate (ThermoFisher, Waltham, MA, USA) and 40 µM of fluorogenic substrate (DMQD-AMC; Sigma, Santa Clara, CA, USA) in 50 µL of lysis buffer was added to each well. The plate was incubated at 37 °C for 2 h and fluorescence was detected using a FluoStar Omega Microplate Reader (excitation *λ* = 355 nm, emission *λ* = 460 nm; BMG, Labtech, East Sussex, UK).

### 4.11. Competitive Protein–Protein Interaction Assay

Pure Hsp70 (1 μg/mL) was added to the wells of a 96-well plate and after blocking with 2 mg/mL BSA in TBS, and then 350 μg/mL of lysate from U-937*wt* cells control and after heat shock (43 °C, 30 min, 6 h of recovery), or U-937*hsp70* that had been treated with 50 μM BT44 and with 2 μM etoposide, was applied to the same wells. The plate was then incubated for 3 h. After washing with TBS with 0.05% Tween 20, anti-Caspase-3 antibody was applied followed by a secondary HRP-conjugated antibody (Abcam, UK). The optical density at 450 nm was measured with the aid of a Fluorofot “Charity” microplate reader (Probanauchpribor, Russia).

### 4.12. Co-Immunoprecipitation

Cultured U-937*wt* cells were incubated with 2 µM etoposide for 4 h and lysed in a buffer containing 20 mM HEPES (pH 7.5), 100 mM NaCl, 1.5 mM MgCl_2_, 0.5 mM EDTA, 0.5 mM DTT, and 0.0025% NP-40. To deplete Hsp70 from the lysates of U-937*wt*, cells treated with etoposide ATP-agarose (Sigma) were used. After depletion of Hsp70, a volume of lysate containing 0.5 mg total protein was incubated for 18 h with anti-Caspase-3 antibody bound to Protein G-Sepharose (Sigma). The beads were washed in the above buffer with 0.05% Tween 20 and transferred to two tubes with 100 µg biotinylated Hsp70 in each, pretreated or not with 32 µM BT44. Mixtures were incubated for 6 h with rotation. After washing, the Sepharose beads were boiled in SDS-sample buffer and subjected to electrophoresis and western blotting with the use of Avidin-peroxidase (Sigma) and anti-Caspase-3 antibody.

### 4.13. Statistics

The data is reported as the mean ± standard errors of the mean (SE). Statistical analysis was performed using one-way ANOVA test supplemented with posthoc test. Differences were considered to be statistically significant when * *p* < 0.05 or ** *p* < 0.01.

## Figures and Tables

**Table 1:** Predicted biological activity spectrum for compound BT44.

Pa	Pi	Activity
0.709	0.003	Caspase 8 stimulant
0.700	0.008	Caspase 9 stimulant
0.542	0.011	Caspase 3 stimulant
0.497	0.014	Heat shock protein 27 antagonist
0.465	0.022	Transcription factor NF κ B stimulant
0.378	0.029	Heat shock protein antagonist
0.387	0.082	Transcription factor NF κ B1 inhibitor
0.334	0.050	Transcription factor NF κ B inhibitor
0.301	0.048	Heat shock protein 90 antagonist

**Table 2:** List of biological activities predicted by PASS 2017 and associated with interaction of drug-like compounds with the selected targets.

Biological Activity	Number	IAP
Caspase 10 inhibitor	6	0.996
Caspase 2 inhibitor	32	0.999
Caspase 3 inhibitor	1112	0.966
Caspase 3 stimulant	115	0.860
Caspase 4 inhibitor	9	0.885
Caspase 6 inhibitor	147	0.999
Caspase 7 inhibitor	937	0.971
Caspase 8 inhibitor	264	0.993
Caspase 8 stimulant	31	0.843
Caspase 9 inhibitor	44	0.968
Caspase 9 stimulant	39	0.779
Caspase inhibitor	7	0.999
Caspase stimulant	28	0.958
Heat shock 70 kDa protein 1 antagonist	16	0.924
Heat shock factor protein 1 inhibitor	271	0.855
Heat shock protein 27 antagonist	9	0.804
Heat shock protein 70 agonist	26	0.904
Heat shock protein 70 antagonist	23	0.965
Heat shock protein 90 alpha antagonist	1016	0.916
Heat shock protein 90 antagonist	1342	0.926
Heat shock protein 90 β antagonist	296	0.983
Heat shock protein HSP 90 (HSC82) inhibitor	11	0.999
Heat shock protein agonist	27	0.883
Heat shock protein antagonist	1374	0.923
Transcription factor NF κ B inhibitor	1009	0.910
Transcription factor NF κ B stimulant	7	0.882
Transcription factor NF κ B1 inhibitor	398	0.866
Transcription factor NF κ B2 inhibitor	74	0.990

Number is the number of compounds with the appropriate activities in the PASS training set; IAP (Invariant Accuracy of Prediction) is the accuracy of prediction estimated in leave-one-out cross-validation.

## Abbreviations

AIF	Apoptosis-Inducing Factor
APAF-1	Apoptotic Protease Activating Factor 1
CMLA	Carboxy-Methylated α-Lactalbumin
DARTS	Drug Affinity Responsive Target Stability
DTT	Dithiothreitol
EDTA	Ethylenediaminetetraacetic Acid
HRP	Horseradish Peroxidase
Hsp70	Heat Shock Protein 70 kDa
IC50	Half Maximal Inhibitory Concentration
JNK	c-Jun N-Terminal Kinase
MST	Microscale Thermophoresis
SAPK	Stress-Activated Protein Kinase
PAGE	Polyacrylamide Gel Electrophoresis
PASS	Prediction of Biological Activity Spectra for Substances
PVDF	Polyvinylidene Difluoride
TBS	Tris-Buffered Saline
